# Tackle Characteristics Resulting in Potential Head Injuries in Elite Men's Rugby League: A Video Analysis Study of 746 Tackles

**DOI:** 10.1002/ejsc.12270

**Published:** 2025-02-25

**Authors:** James Woodward, Ben Jones, Gemma Phillips, Kevin Till, Sharief Hendricks, Ross Tucker, Chris Bleakley, Gregory Tierney

**Affiliations:** ^1^ Sport and Exercise Sciences Research Institute Ulster University Belfast UK; ^2^ Carnegie Applied Rugby Research (CARR) Centre Carnegie School of Sport Leeds Beckett University Leeds UK; ^3^ England Performance Unit The Rugby Football League Mancheser UK; ^4^ Division of Physiological Sciences Department of Human Biology Faculty of Health Sciences University of Cape Town Cape Town South Africa; ^5^ Faculty of Health Sciences School of Behavioural and Health Sciences Australian Catholic University Brisbane Australia; ^6^ Premiership Rugby London UK; ^7^ Uno‐X Cycling Oslo Norway; ^8^ Leeds Rhinos Rugby League Club Leeds UK; ^9^ World Rugby Dublin Ireland; ^10^ School of Health Science Ulster University Belfast UK; ^11^ School of Engineering Ulster University Belfast UK

**Keywords:** head contact, head injuries, rugby, tackling

## Abstract

**Objectives:**

Contact with the head should be avoided during a rugby league tackle, given the inherent risks of head injuries. This study aimed to characterise a sample of tackles, retrospectively identified as resulting in a potential head injury by the Rugby Football League (RFL) match review panel.

**Design:**

Retrospective video analysis study.

**Methods:**

746 tackles, identified by the RFL match review panel from the men's 2018 and 2019 Super League seasons, were analysed. Video clips were coded using an adapted analysis framework, characterising tackle stage, head contact, affected player, offending player/surface, offending body part/surface and tackle sanctioning. Data were reported as frequencies and percentages.

**Results:**

The majority of tackles resulting in a potential head injury occurred in the initial tackle contact stage (*n* = 590, 79.2%). The ball‐carrier was most frequently affected (*n* = 372, 49.9%) compared to initial tacklers (*n* = 213, 28.6%). The initial tackler was the most frequently impacting player (*n* = 268, 36.0%), with the majority of potential head injuries occurring from direct head contact by the arm (*n* = 230, 34.1%), shoulder (*n* = 170, 25.2%) and head/neck (*n* = 145, 21.5%) of the impacting player. Head contact was present in 90.6% (*n* = 675) of the tackles resulting in a potential head injury. Of the sample of tackles, 16.1% (*n* = 109) of direct head contact events received a sanction from on‐field match officials.

**Conclusion:**

The initial tackle contact between the ball‐carrier and initial tackler remains the area of focus for research into potential head injuries in elite‐level men's rugby league, to improve awareness and understanding of the mechanisms of injury.

**Summary**

- The initial tackle contact was observed to be the main area of the tackle that results in a potential head injury in this sample (79.1%), with the ball‐carrier being the most frequently affected player and the initial tackler being the most impacting player.
- Direct head contact to the affected player was observed in the majority of tackles (90.6%) resulting in a potential head injury, primarily as a result of contact by the upper body (arm, shoulder, head/neck) of the impacting player.
- In the sample of tackles analysed, a low rate of sanctioning of direct head contact events that resulted in a potential head injury was reported (16.1%). Further consideration needs to be given to understand factors that may influence sanctioning rates.

## 1 Introduction

Rugby league is a contact sport, where players are involved in approximately 13–25 tackles per match (Naughton et al. 2020). The tackle is a key element of the game, however, it is also the most injurious event during a match (Hopkinson et al. 2022a; King et al. 2010a; Gardner et al. 2015a, 2021; S. Hendricks et al. 2021). One injury outcome of concern during the tackle is a head injury, such as concussion, which can occur because of direct contact with a player's head or through indirect head loading (Hinton‐Bayre, Geffen, and Friis 2004). Studies investigating the concussion incidence in rugby league have highlighted an incidence range of 14.9 concussions per 1000 player‐match‐hours in the Australasian National Rugby League (NRL) to 15.5 across the 2016 to 2022 playing seasons in the European Super League (ESL) (Eastwood et al. 2023; A. J. Gardner et al. 2017). Understanding the characteristics of potentially injurious tackles can help identify high‐risk actions, which can be used to provide guidance on how to mitigate their occurrence (Finch 2006).

Whilst research into head injury event characteristics in the tackle event have been explored and identified in rugby union, these have yet to be explored to the same degree in rugby league (Tucker et al. 2017a; Tierney et al. 2018a, 2018b). In addition, characteristics of head injuries between rugby codes may not be comparable, because of technical differences in laws and outcomes of tackles. For example, in rugby union a tackle can lead to a competed ruck defined as forming “when at least one player from each team is in contact, on their feet and over the ball” (World Rugby 2023a). In rugby league, a tackle results in a wrestle to the floor or until held, followed by a play of the ball (M. Hopkinson et al. 2022b; National Rugby League; Rugby Football League). There are also differences in tackle approaches and outcomes between rugby codes. In rugby league the players have 6 set phases to gain field territory, whereas rugby union has no set number of phases. These technical differences may also cause difficulty in translating law trials and considerations from rugby union to rugby league, emphasising the need for rugby league specific research and interventions. By analysing the characteristics of tackles that result in a potential head injury in elite men's rugby league, more accurate law modifications and interventions can be proposed to improve player safety in the elite men's competition. Given the inherent risk of head injuries in rugby league, and the growing body of evidence of their potential long‐term effects on players, rugby league needs to continue to undertake research to prioritise player welfare (A. Gardner et al. 2015a; McMillan et al. 2017).

Video analysis has been previously utilised as a tool for identifying characteristics of tackles that result in head contact or head injury (Gardner et al. 2015b, 2017; Hendricks et al. 2020). Video analysis has also been used by previous studies to identify potential risk factors for head injuries in rugby union and rugby league (Gardner et al. 2015b, 2017; Tucker et al. 2017a, 2017b). The results from these studies have then been considered to develop law interventions across different levels of the sport (World Rugby 2023b; Football League). Studies investigating head contact in rugby league have utilised a combination of video analysis and analysis frameworks to identify particular head injury risk factors in player cohorts, such as shoulder charge tackles (King et al. 2010a; Cummins et al. 2015; Gabbett et al. 2005; Hoskins et al. 2006). Additional studies have also explored differences in who is more at risk of head injury, identifying the tackler to have a greater propensity for head injury in both elite men's (NRL) and women's rugby league (NRL and ESL) players (Gardner et al. 2015a, 2021; Spiegelhalter et al. 2023; McLeod et al. 2023). However, further research is needed within the elite men's ESL cohort to draw comparisons between playing competitions. Therefore, the importance of analysing and understanding the characteristics of such tackles is clear.

In addition, contact sports involving a tackle (e.g., rugby union and rugby league) have head contact laws in place to prevent players from making contact with another player's head, in order to reduce the potential risk of a head injury. In the ESL competition, if the tackler “makes contact with the head or neck of an opponent intentionally, recklessly or carelessly”, this would be deemed in violation of those rules (Rugby Football League). This could then result in an on‐field sanction, in the form of a penalty, sin bin or sending off, with the potential for further citations post‐match. Despite such laws, tackles that result in head contact resulting in a potential head injury can still occur during a match and pose a risk to the ball‐carrier and tackler. This study therefore aimed to utilise a combination of video analysis and adapted rugby analysis frameworks, to explore risk factors in a sample of tackles resulting in a potential head injury in the 2018/19 seasons of the elite men's ESL. This study also aimed to explore sanctioning of these potential head injury tackle events.

## 2 Materials and Methods

This study retrospectively analysed a sample of 825 events identified by the Rugby Football League (RFL) match review panel (MRP), from the 2018 and 2019 European Super League seasons, identified as resulting in a potential head injury. One of the roles of the MRP is to review match footage retrospectively and identify, clip and store incidents and events per match which may have resulted in a potential head injury to a player. These are then collated and reviewed throughout the playing season. The MRP consists of the RFL Compliance Manager, four retired professional players, coaches and a match official (Football League).

The first author (JW) subsequently analysed the 825 video clips. Video clips with poor video quality and occlusion of the tackle or event were removed (*n* = 79). After this initial screening, 746 clips remained for further analysis. Ethics approval for this study was given by Leeds Beckett University Local Ethics Review Committee (#78482).

Video clips included 30–50 s of footage highlighting the pre‐contact, during and post‐contact event, allowing for a full analysis of the mechanisms in the build‐up to the tackle and post‐contact. Video footage was of broadcast quality and captured at 25 frames per second with some clips containing slow‐motion broadcast replays and multiple viewing angles. The first author (JW, 3 years' experience of rugby video analysis) analysed the clips using a frame‐by‐frame analysis in Kinovea video editing software. This approach reduced multi‐coder variance in reporting (Tucker et al. 2017a). The coder was able to view the video clips as many times as necessary.

The included video clips were then coded using an analysis framework (Table 1) adapted from Tucker et al. (2017a), M. Hopkinson et al. (2022b) and Hendricks et al. (2020). Tackle events were initially coded by the stage of the tackle; ‘initial tackle contact’, ‘secondary tackle contact’, ‘play the ball phase’, ‘head to ground’, or ‘other’ (Figure S1). ‘Other’ was defined as any event resulting in a potential head injury outside of the above events (e.g., ball to head, celebrations, off the ball incidents). Tackle events were then coded for the impact mechanics; direct/inertial head loading, the role of the impacting (e.g., the player responsible for making contact with another player's head) or affected (e.g., the player whose head is made contact with) player, impacting body part/surfaces involved and sanctioning of direct head contact events. Results are presented as percentages and count of events.

**TABLE 1:** Impact Analysis Framework with variables coded and their components and definitions, with Cohen's kappa scores for inter and intra‐rater reliability.

Characteristic	Cohen's K (Cohen, 1960)	Coded factors and definitions
Tackle stage Hopkinson et al. (2022b, 2022a)	Intra 0.95 Inter 0.82	Initial tackle contact – The event occurs from the first collision made between the ball carrier and each unique tackler Secondary tackle contact – The event occurs after the initial collision between the same ball carrier and tackler has been made and before the ball carrier is grounded Play the ball phase – The event occurs after the ball carrier has been grounded before a new phase begins Ground impact – An event caused by players falling to the ground during a tackle; forces can be transmitted through the player Other – Any other event, outside of the above events (e.g., ball‐to‐head, celebrations, off‐the‐ball incidents)
Head contact	Intra 0.95 Inter 0.92	Present – Direct contact with the head of the affected player Absent – Indirect/inertial head loading transmitted through the neck from an impact to the body
Affected player	Intra 0.95 Inter 0.94	Ball carrier (BC1) – The affected player is in possession of the ball Initial tackler (T1) – The affected player is the initial tackler, attempting to stop the ball carrier Secondary tackler (T2) – The affected player is a second tackler, attempting to aid the initial tackler in stopping the ball carrier Additional tackler (T3+) – The affected player is any extra tackler(s) joining the initial and secondary tackler to stop the ball carrier Other – Any affected player that is involved in off‐the‐ball or aerial collisions
Offending player/surface Tierney et al. (2018a)	Intra 0.94 Inter 0.91	Ball carrier (BC1) – Offending player is in possession of the ball Initial tackler (T1) – Offending player is the initial tackler attempting to stop the ball carrier Secondary tackler (T2) – Offending player is the secondary tackler attempting to aid the initial tackler in stopping the ball carrier Additional tackler (T3+) – Offending player is the tertiary or extra tackler attempting to aid the initial and secondary tacklers in stopping the ball carrier Ground – The event was a direct consequence of head‐to‐ground impact Other – Offending player involved in off‐the‐ball or aerial collisions where the ball is not involved
Offending body part/surface Hendricks et al. (2020)	Intra 1.00 Inter 0.95	Lower leg – Offending body part was the lower leg, from knee to foot Hip – Offending body part was the hip Upper leg – Offending body part was the upper leg, from above the knee to below the hip Torso – Offending body part was the torso Shoulder – Offending body part was the shoulder Arm – Offending body part was the arm Head/Neck – Offending body part was the head/neck area Ground – The ground was the offending surface N/A – Non‐direct impacts to the body with no offending body part or surface involved
Sanctioning of contact Hendricks et al. (2020)	Intra 1.00 Inter 1.00	Unsanctioned – Contact deemed legal by match officials receiving no sanction (e.g.: Penalty, sin bin, sending off) Sanctioned – Contact deemed illegal by match officials, receiving a sanction (e.g.: Penalty, sin bin, sending off)

To assess inter‐rater reliability, an external reviewer (Rugby Performance Analyst) analysed a randomly selected sample of 25 direct head contact video clips using the same analysis framework established for this study. Reliability was tested using Cohen's kappa (k) in SPSS (Cohen 1960). Intra‐rater reliability was similarly tested with the original coder (JW) reviewing the same 25 video clips at 1 month post initial review to retest reliability using Cohen's kappa (k) (Cohen 1960). Cohen's kappa values for each analysed variable can also be found in Table 1, where a Cohen's kappa value of over 0.80 indicates near‐perfect agreement (Cohen 1960). This study reported the proportions of tackle events that resulted in a potential head injury by the analysed variables established in Table 1. All data analysis was undertaken in MATLAB and GraphPad.

## 3 Results

Table 2 below shows the percentage of events resulting in a potential head injury, in each coded tackle stage. Overall, the initial tackle contact accounted for the greatest proportion of all tackle events resulting in a potential head injury (*n* = 590, 79.1%), followed by secondary tackle contact (*n* = 61, 8.2%).

**TABLE 2:** Descriptive statistics (*n*, %) of tackle stage.

	Initial tackle contact	Secondary tackle contact	Ground impact	Play the ball phase	Other
Count of events	590	61	18	29	48
Percentage of total events	79.1%	8.2%	2.4%	3.9%	6.4%

Table 3 displays the proportion of tackles resulting in a potential head injury for each player role. The ball carrier was more frequently affected than any other player (*n* = 372, 49.9% vs. initial tackler (T1) *n* = 213, 28.6%, secondary tackler (T2) *n* = 99, 13.3%, additional tackler (T3+) *n* = 27, 3.6% and other *n* = 35, 4.7%)).

**TABLE 3:** Descriptive statistics (*n*, %) of role of affected player.

	BC1	T1	T2	T3+	Other
Count of events	372	213	99	27	35
Percentage of total events	49.9%	28.6%	13.3%	3.6%	4.7%

Table 4 below shows the proportion of tackles resulting in a potential head injury for each impacting player role. The initial tackler (T1) was the most frequently impacting player compared to any other player role (*n* = 268, 36.0% vs. ball carrier (BC1) *n* = 201, 27.0%, secondary tackler (T2) *n* = 165, 22.1%, additional tackler (T3+) *n* = 52, 7.0%, other *n* = 33, 4.4% and ground *n* = 27, 3.6%).

**TABLE 4:** Descriptive statistics (*n*, %) of role of impacting player or surface.

	BC1	T1	T2	T3+	Ground	Other
Count of events	201	268	165	52	27	33
Percentage of total events	27.0%	36.0%	22.1%	7.0%	3.6%	4.4%

**FIGURE 1:**
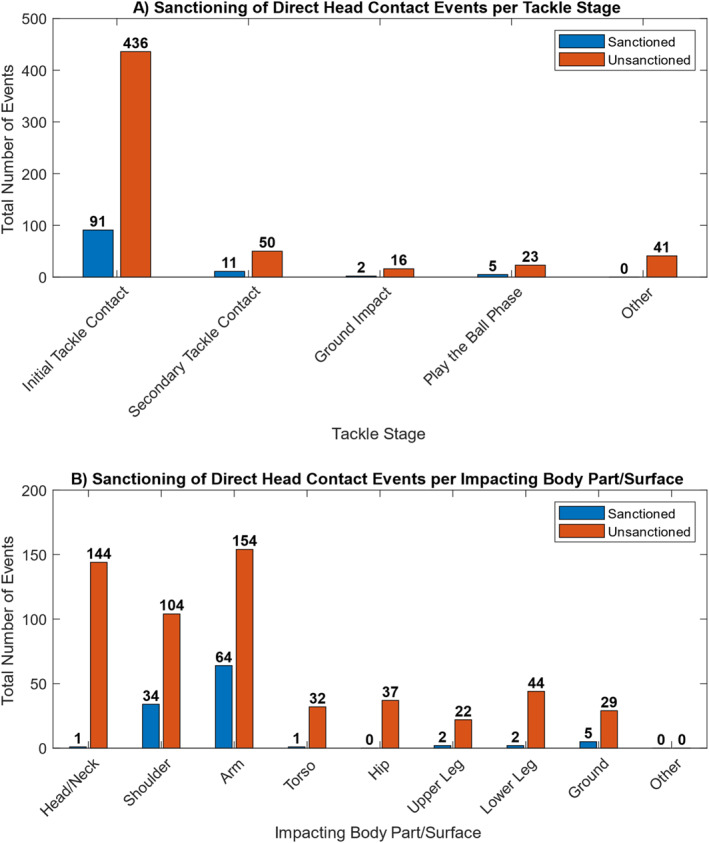
(A) Sanctioning of direct head contact events by tackle stage, (B) sanctioning of direct head contact events by the impacting body part/surface.

Direct head contact was observed more frequently than inertial head loading in tackles resulting in a potential head injury (*n* = 675, 90.6% vs. *n* = 70, 9.4% of all tackle events). The initial tackle contact stage accounted for the majority of all direct head contact events (*n* = 527, 78.1% vs. secondary tackle contact (*n* = 61, 9.0%), play the ball phase (*n* = 28, 4.1%), ground impact (*n* = 18, 2.7%) and other (*n* = 41, 6.1%)). Figure 1A shows the proportion of direct head contact tackle events in each tackle stage and the on field sanction decision (sanctioned or unsanctioned) given by the match official. Of the direct head impacts observed in this study, 16.1% (*n* = 109) received a sanction by match officials on the field. Of these 16.1%, direct head contact in the initial tackle contact (*n* = 91, 83.5%) received the most sanctions, followed by secondary tackle contact events (*n* = 11, 10.1%), play the ball phase (*n* = 5, 4.6%), and ground impact (*n* = 2, 1.8%).

Figure 1B displays the sanctioning of direct head contact events, broken down by the impacting body parts/surface in the tackle events. The majority of direct head contact events occurred as a result of contact to the head by the arm (*n* = 218, 32.3%), shoulder (*n* = 138, 20.4%), and head/neck (*n* = 145, 21.5%) of the impacting player, with 15.9% because of the lower body (hip (*n* = 37, 5.5%), upper leg (*n* = 24, 3.6%), lower leg (*n* = 46, 6.8%)) and the remainder because of the torso of the impacting player (*n* = 33, 4.9%) and the ground (*n* = 34, 5.0%). The upper body (arm, shoulder, and head/neck) was responsible for the majority of all direct head contact events (*n* = 501, 74.2%), with arm, shoulder and head/neck to head events receiving a combined sanction frequency of 14.7% (*n* = 99) by the on‐field match official.

## 4 Discussion

This study examined the characteristics of tackles resulting in a potential head injury from a sample of tackle events from the 2018 and 2019 ESL playing seasons. Descriptive analysis of the tackle events revealed that 79.1% (*n* = 590) of the tackles resulting in a potential head injury occurred in the initial tackle contact, suggesting that the initial tackle contact remains the main area of the tackle that results in a potential head injury. Contrasting to research in the elite men's NRL competition, in this study almost half of the potential head injuries occurred to the ball‐carrier (49.9%), followed by the initial tackler (28.6%) (Gardner et al. 2015a, 2015b, 2021). The initial tackler was also the player most frequently causing the potential head injury (36.0%). Direct head contact to the affected player was observed in the majority (*n* = 675, 90.6%) of tackle events in this sample, as a result of contact by the arm, shoulder or head/neck of the impacting player. In the initial tackle contact, the initial tackler more frequently caused direct head contact with the ball carrier by striking with their arm (37.1%) and shoulder (33.5%). In the same tackle stage, the initial tackler was exposed to direct head contact more frequently by the arm (31.1%) or head/neck and shoulder (17.7%) of the ball carrier. Furthermore, a low proportion of direct head contact events received a sanction on field by match officials (16.1%). Therefore, whilst a more stringent stance on officiating direct head contact is key, improving player tackle technique through interventions should be considered in tandem. Engaging all stakeholders (e.g., referees, coaches, players) in improving player safety may be advantageous to reduce the number of rugby league tackles that result in head contact or potential head injury. Previous research has also highlighted that a unified approach across stakeholders can lead to more effective law and coaching interventions (N. Burger et al. 2020).

The majority of tackle events resulting in a potential head injury were because of direct head contact with the affected player (*n* = 675, 90.6%). Identifying strategies to reduce this occurrence may benefit rugby league. Additionally, head injuries occurring as a result of direct head contact are reportedly more likely to be identified by medical staff than inertial head loading causes (Savage et al. 2013). As head loading can also occur through inertial loading, in addition to the tackle events that result in direct head contact, it is important to consider the tackles that did not have direct head contact (*n* = 70, 9.4% of the sample analysed) and further attention should be given to avoid non‐removal of players exhibiting head injury symptoms without an observed direct head contact (Elkin, Elliott, and Siegmund 2016). The additional risk of repeated head contact resulting in head injuries to players continuing to play despite temporary or unrecognised symptoms is also well documented (Savage et al. 2013). Removal of the player for assessment following any contact event resulting in a potential head injury from either direct or indirect head loading is therefore paramount, because of the potential cumulative negative effect of concussions, in the short‐ and long‐term (Gronwall et al. 1975).

In the sample of tackles analysed, 16.1% (*n* = 109) of direct head impact events received a sanction on‐field from match officials. The low number of tackles that received an on‐field sanction may be because of the sample of video clips identified by the match review panel which may not be representative of all head contact sanctioning rates in the ESL. Despite this, the low rate of sanctioning of direct head contact tackles is important given that illegal and high tackles are more likely to result in a head injury (Davidow et al. 2018; Hendricks et al. 2015). The tackle event sample included in this study is from the 2018 and 2019 European Super League season, where 141 and 180 matches were played respectively (total of 321 matches across both seasons). The video clips of direct head contact which were unsanctioned by the on‐field match official (*n* = 566) were representative of approximately two per match over the two seasons. It is important to note, however, that since these playing seasons a review of sanctioning of direct head contact events has been undertaken, with stricter head contact law trials being implemented and a decision‐making framework for match officials developed (Raftery, Tucker, and Falvey 2021; Stokes et al. 2021). Furthermore, the 746 video‐verified events analysed in this study are representative of only a small sample of the > 150,000 tackle events which occurred during these seasons (Rennie et al. 2022). Consequently this sample may not include all potential head injury events that occurred over these playing seasons (Rennie et al. 2022). However, the low sanctioning rates remain a cause for concern and raise key questions around officiating and player behaviours on field. Further research should aim to work alongside match officials, coaches, and players to explore potential player behavioural interventions as well as officiating interventions to improve player safety.

Reducing the frequency of head contact in the game is not solely the role of the match officials. Coaching, delivering and executing safe tackle technique is also the responsibility of the players, coaching staff and governing bodies of the sport (Davidow et al. 2018; Hendricks et al. 2015). Previous studies have highlighted a high incidence rate of potential head injuries during illegal play, which also highlights the importance of shared responsibility in player safety management (Hinton‐Bayre, Geffen, and Friis 2004). Given that this study found that the majority of events resulting in a potential head injury were because of the arm, shoulder, or head/neck impacting the head of the affected player, improving tackle technique would be beneficial to improving player safety. For example, this may include encouraging tackling players to bend at the waist given that upright tackles have a higher propensity for head contact in both rugby league and rugby union (A. J. Gardner et al. 2021; Tucker et al. 2017a). Studies investigating the proficiency of tackles identified that safer tackles (non‐head impacting tackles) were also associated with better tackle proficiency scores (Davidow et al. 2018; Hendricks et al. 2015; Burger et al. 2016). It is important to note however, that in contrast to rugby union, a major outcome goal for the tackle in rugby league is to prevent the attacker from offloading the ball because of a finite number of tackles in a set (set of 6) (D. King et al. 2010b; Speranza et al. 2018). Therefore, preventing the offload of the ball is key, to force the attacking team to use up their allowed phases, as such, players target their tackle around the chest and ball (D. King et al. 2010b; Speranza et al. 2018). Reducing the tackle height, such as trialled in rugby union (World Rugby 2023b), may disadvantage the defending team in this regard. Given this tactical difference between codes, future studies should research a tactically advantageous tackle height for attackers and defenders specific to rugby league, without compromising on player safety. In the meantime coaches should prioritise coaching good tackle technique, which when implemented by players, can result in safer and also more successful (from a performance perspective) tackles.

This study reviewed video footage of players who were identified as sustaining a potential head injury. Similar to other studies, the clinical outcome of whether the player sustained a head injury and the degree of the injury was not made available (Tierney et al. 2018b). Furthermore, knowledge of which players were removed from the field following the potential head injury was also not available. Without knowledge of the clinical outcome, it is difficult to determine whether the players were either removed temporarily or permanently from play. It is also difficult to determine whether they returned to play in the same game or even presented with latent symptoms post‐match. Without this knowledge it is also unknown as to whether the characteristics of these tackle events vary by degree of severity of head injury. The study is descriptive in design, though future work looking at injury risk and propensity is warranted for the development of player protection strategies.

A total of 746 out of 825 video clips of head impact events were included in this study, with 79 (9.6%) being discounted because of poor video quality or occlusions. Impact events that were filtered out may have provided additional insight into the events leading to a potential head injury in the current study. The video clips were all from match play and, as such, did not include any head injuries that may have occurred during training sessions. These warrant future investigation because of potential differing injury mechanisms (Gabbett 2004). Future video analysis studies should compare the characteristics of tackles in both matches and training in elite and amateur rugby league. In addition, girls' and women's amateur and elite rugby league remain an under‐researched area. It is important to explore women‐specific rugby league law interventions given early research has identified some differences in incidence and mechanism (Spiegelhalter et al. 2023; McLeod et al. 2023). Other tackle analysis variables should also be explored in future research, such as fatigue and player tackle count, in order to explore wider factors influencing potential head injury risk (Gabbett 2008, 2016). Finally, the magnitude of the head impact needs further investigation via the measurement of head kinematics, which can be monitored by the inclusion of validated instrumented mouthguards (Jones et al. 2022).

## 5 Conclusion

The initial tackle contact remains the main area of the tackle that results in a potential head injury. The ball carrier was the most frequently affected player in the sample, followed by the initial tackler who was also the most frequently impacting player. Direct head contact to the affected player was observed in the majority of tackle events analysed, more frequently associated with contact by the arm, shoulder or head/neck of the impacting player, with a low rate of sanctioning by on‐field match officials. The findings from this study provide a focus for future research into player protection strategies and support for match officials in sanctioning.

## Conflicts of Interest

S.H. is Social Media Editor and an Associate Editor for EJSS. S.H. is also a research consultant for World Rugby. B.J. is employed in a consultancy capacity by the Rugby Football League and Premiership Rugby. G.P. is also the Deputy Chief Medical Officer of the Rugby Football League.

## Supporting information

Supplementary Material

## Data Availability

The data that support the findings of this study are available upon reasonable request to the corresponding author, J.W.
