# Supplementary material for: Tackle Characteristics Resulting in Potential Head Injuries in Elite Men's Rugby League: A Video Analysis Study of 746 Tackles
Source: Eur J Sport Sci. 2025 Feb 25;25(3):e12270. doi: 10.1002/ejsc.12270 (PMC11855370; doi:10.1002/ejsc.12270)
Supplement: Supplementary file 1 — Supplementary Material [file EJSC-25-e12270-s001.docx]

**Supplementary Figure 1**


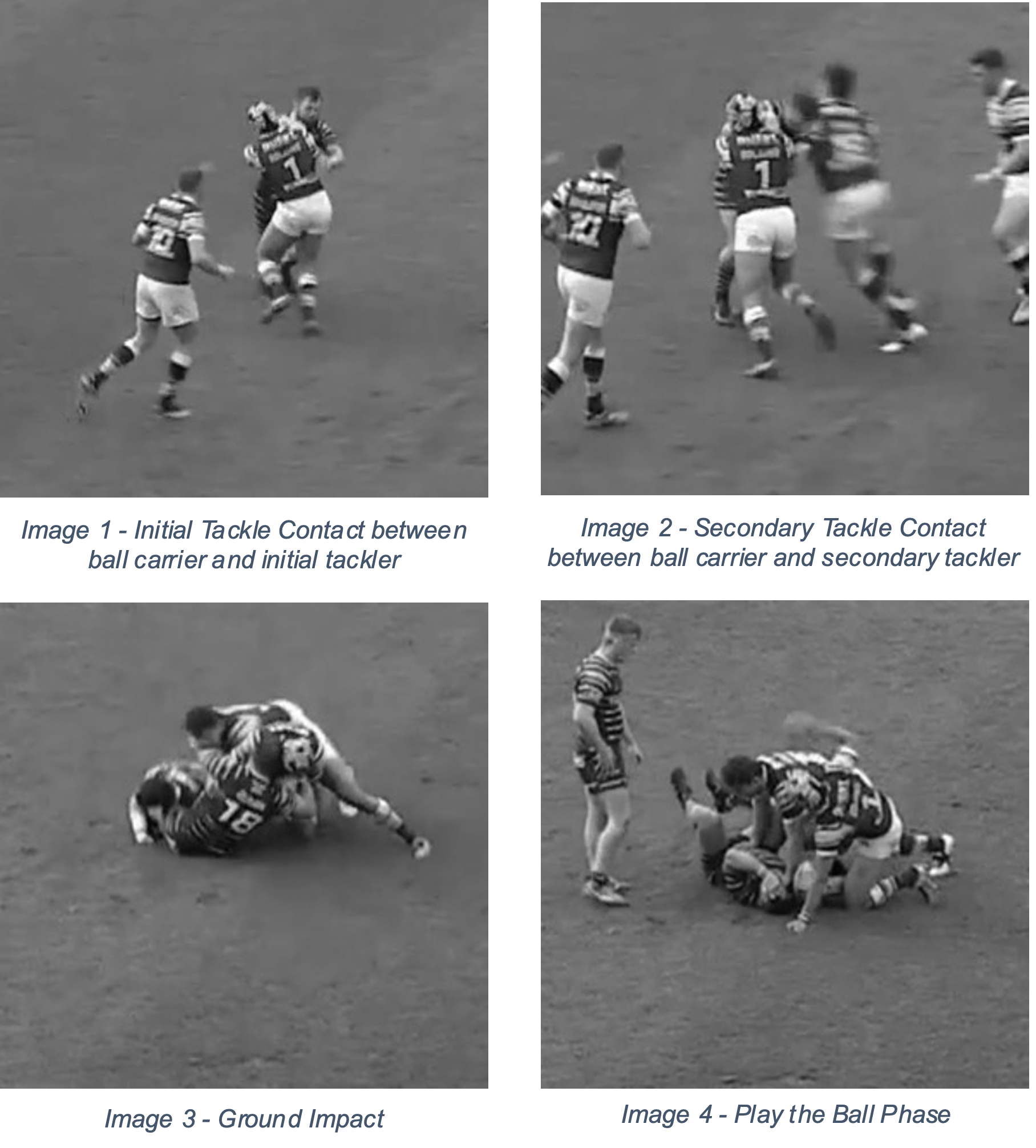


**Supplementary Fig 1:** Still images recorded from video footage, greyed out and blurred for player anonymity. Images depict the coded Tackle Stages; Image 1 – Initial Tackle Contact, Image 2 – Secondary Tackle Contact, Image 3 – Ground Impact and Image 4 – Play the Ball Phase.
